# Computerized decision support for antimicrobial prescribing: what every antibiotic steward should know

**DOI:** 10.1017/ash.2025.10091

**Published:** 2025-09-12

**Authors:** Davide Bosetti, Rebecca Grant, Gaud Catho

**Affiliations:** 1 Infection Control Programme and WHO Collaborating Centre for Infection Prevention and Control and Antimicrobial Resistance, Geneva University Hospitals and Faculty of Medicine, Geneva, Switzerland; 2 Division of Infectious Diseases, Central Institute Valais Hospital, Sion, Switzerland

## Abstract

**Objective:**

To examine the potential role of computerized clinical decision support systems (CDSS) in antimicrobial stewardship (AMS) and to identify significant challenges concerning their effectiveness and implementation.

**Design:**

Narrative review.

**Setting and methods:**

This review is based on existing literature regarding CDSS in AMS across various healthcare environments, such as hospitals and primary care facilities. The systems evaluated include both stand-alone tools and those integrated into electronic health records (EHR), featuring expert rule-based logic and new machine learning (ML) models. CDSS capabilities include prescribing guidance, alerts, resistance prediction, and de-escalation protocols.

**Results:**

CDSS are intended to aid in antimicrobial prescribing by integrating clinical guidelines with data specific to each patient. Despite their theoretical potential, their effectiveness is often hindered by inconsistent incorporation into clinical practices, low user engagement, and inadequate design. Many systems are reactive, not well-suited to user needs, or lack transparency in their recommendations. Evaluating these systems is challenging due to varied outcomes, poor methodological quality of studies, and the complexity of attributing causality in intricate care settings. Barriers to implementation include alert fatigue, perceived time constraints, poor fit with existing workflows, and resistance to change. Instances like the COMPASS trial demonstrate the disconnect between design and practical application, underscoring the necessity for user-focused development, clear reasoning, and a balanced approach between mandatory and advisory elements.

**Conclusions:**

CDSS have the potential to improve antimicrobial use, but widespread impact is hindered by evaluation and implementation challenges. Realizing their value requires better integration, usability, and rigorous research frameworks tailored to complex healthcare settings.

## Background

Antimicrobial resistance (AMR) is a complex public health issue. A modeling study estimated that 4.95 million deaths globally in 2019 were related to bacterial AMR, with a disproportionate impact on resource-limited areas.^1^ Inappropriate use of antimicrobials remains a key driver of AMR, particularly in outpatient settings and hospitals. For example, 25–28% of antibiotic prescriptions for respiratory infections in ambulatory care are unnecessary,^2^ while up to 50% of hospital antibiotic use is considered inappropriate.^3^

To preserve the effectiveness of antimicrobial agents, it is imperative to improve the use of antimicrobials globally.^3^ Antimicrobial stewardship (AMS) programs aim to optimize antimicrobial prescription by selecting the appropriate agent, dose, and duration tailored to individual patients.^4^ AMS strategies include clinical guideline adherence, de-escalation, intravenous-to-oral switches, drug monitoring, and specialist consultation.^5^ These interventions have been associated with reduced antibiotic use,^6^ but are often resource-intensive, cover only a subset of prescriptions, and are typically confined to regular working hours.^7^ Moreover, they are usually reactive, implemented after antimicrobials have already been prescribed, potentially missing opportunities to prevent unnecessary exposure.^8,9^

Computerized clinical decision support systems (CDSS) are software designed to support healthcare professionals in clinical decision-making. For AMS interventions, CDSS can overcome limitations of “human” interventions by overseeing antibiotic prescriptions in electronic health records (EHR) continuously. CDSS function as proactive support systems, assisting in decision-making before antimicrobial prescription.^8^ CDSS integrate with EHR data and incorporate key AMS components like preauthorization, duration suggestions, medication switches, de-escalation strategies, allergy alerts, reassessment protocols, and restrictions, enabling multimodal interventions.

In this review, we aim to summarize current evidence available on CDSS as tools for AMS interventions, with a focus on evidence of their effectiveness and challenges to implementation.

### Computerized clinical decision support systems in healthcare

CDSS offer evidence-based recommendations and information relevant to patient care. CDSS combine patient data, medical knowledge, and clinical guidelines, enabling healthcare providers to effectively diagnose diseases, choose appropriate treatments, and ultimately improve patient outcomes.^10^ Originally developed for academic research in the 1970s, CDSS have evolved to include stand-alone applications as well as tools integrated into EHR and computerized provider order entry (CPOE) systems.^10^

CDSS can be classified into two types: expert systems and machine-learning (ML) systems.^11^ The former, which constitutes the majority, so far operates based on rules established according to expert opinion and guidelines. In these systems, the quality of the experts’ knowledge and recommendations is crucial. In contrast, ML-based systems use algorithms—including supervised and unsupervised learning methods, such as neural networks and deep learning—to identify patterns and improve predictions based on available data. The performance of these systems depends on the quality, completeness and relevance of the input data, which directly influences the accuracy and validity of the outputs. Unlike expert-based systems, ML algorithms continuously learn from new data and adapt over time, allowing for dynamic, real-time insights into complex prescribing scenarios.^11^ The integration of ML into AMS advances the development of precise, predictive decision support systems. These models are able to synthesize a wide range of extensive datasets—including clinical history, microbiology results, pharmacy data, and genomics—to predict AMR profiles and optimize empiric therapy. ML-based CDSS remain mostly experimental. Systems used in real settings like the Veterans Health Administration (VA), UK National Health Service (NHS), or Epic modules still rely on expert-rule-based algorithms rather than adaptive machine learning.^12^ The development of ML techniques and the interoperability of ML algorithms with EHR data may be used to optimize the use of antimicrobials among patients. ML algorithms can predict individual infection resistance profiles, improving the appropriateness of empiric therapy even before culture and susceptibility results—traditionally several days—are available.^13^ Table 1 highlights three recent examples of ML-driven CDSS designed to improve antimicrobial prescribing through personalized antibiotic resistance prediction and empirical treatment selection.

**Table 1.** Examples of ML CDSS for antimicrobial prescribing

First Author, Year	Infection type	ML model type and data inputs	Outcomes (by category)
**Yelin et al., 2022** ^42^	Outpatient UTI	ML algorithm (demographics, past urine cultures, antibiotic history)	- Therapy optimization: Reduced empirical mismatches from >8% to <6%
**Kanjilal et al., 2020** ^43^	Outpatient UTI	ML algorithm (demographics, clinical history)	- Antibiotic use reduction: 67% reduction in second-line use - Therapy optimization: 18% decrease in inappropriate prescribing
**Stracy et al., 2022** ^44^	Inpatienturinaryandwound infections	ML analysis (whole-genome sequencing of clinical isolates)	- AMR prevention: Identified personalized regimens to minimize resistance emergence

ML: machine learning; UTI: urinary tract infection; AMR: antimicrobial resistance.

Despite these promising advancements, many ML-based CDSS have so far demonstrated performance largely through internal validation on retrospective datasets, with limited external validation and prospective evaluation, both of which are needed for clinical implementation. ML implementation is limited by access to quality annotated data, local microbiological variations, and integration into clinical workflows.^14,15^ There may also be issues related to biases in training and test data set used to develop ML models, as well as limited interpretability—clarifying the relative contributions of various features to the models—or explainability—showing how the models generate their outputs and reach their conclusions—of ML models which may be a barrier to their adoption in clinical settings.^16^ The continued development of artificial intelligence and ML techniques is expected to significantly impact AMS interventions in the short to medium term. Hybrid models integrating expert rules with machine learning may offer an optimal solution by enhancing interpretability while maintaining prediction. The use of large datasets, for example through collaborations with national, or linked health data systems could also be helpful for assessments of effectiveness.

### Modes of implementation for AMS digital tools :“stand-alone” tools versus CDSS integrated within EHR

Digital tools for AMS can be implemented as standalone systems or as CDSS integrated into EHR, as shown in Table 2. Stand-alone tools, typically accessed via mobile devices, function independently of EHR, while integrated tools are embedded within EHR systems and use patient-specific data for decision support.^17^ Stand-alone smartphone applications provide clinicians quick access to antimicrobial therapy guidance from local to international sources. These applications offer accessibility, frequent updates, and comprehensive pathogen and treatment information at lower costs than integrated systems. However, their effectiveness depends on clinicians’ usage, and data source quality must be verified.^18^

**Table 2.** Examples of stand-alone tools versus integrated CDSS for AMS

Stand-alone tool name	Country	Custom guidelines	Fixed guidelines	Type of subscription	Subscriber type	Platform	Guidelines sharing	Evidence summary^+^
FirstLine Clinical Decision®	Canada	✓	✘	Fee-based for institutions, Open Access for end-user	Institution	iOS Android Web	✓	3 peer-reviewed studies
Antibioclic®	France	✘	Yes, based on national guidelines of France	Free	Single user	Web	✘	3 observational studies
Sanford Guide’s Antimicrobial Stewardship®	USA	✓	✓	Fee-based	Single user or Institution	iOS Android Web	✘	No peer-reviewed studies
Johns Hopkins ABX Guide®	USA	✘	✓	Fee-based	Single user	iOS Android Web	✘	No peer-reviewed studies
UpToDate®	USA	✘	✓	Fee-based	Single user or Institution	iOS Android Web	✘	3 peer-reviewed studies
Clinical Key®	UK	✘	✓	Fee-based	Mostly institution but single user possible	iOS Android Web	✘	No peer-reviewed studies
DynaMed®	USA	✘	✓	Fee-based	Single user or institution	iOS Android Web	✘	No peer-reviewed studies

Integrated tool name	Country	Alert system^*^	Dynamic reports^*^	Dose-checking^*^	IV-PO switch^*^	IPC module^*^	Evidence summary^+^
Nosotech® : Antibiokos	Canada	✓	✓	✓	✓	✓	No peer-reviewed studies
Epic Systems Corporation®: antimicrobial stewardship module (EASM)	USA	✓	✓	✓	✓	✓	4 peer-reviewed studies
Oracle Health® (fomerly Cerner)	USA	✓	✓	✓	✓	✓	3 observational studies
TheraDoc by Premier Inc.’s®	USA	✓	✓	✓	✓	✓	2 peer-reviewed studies
Vecna QC Pathfinder by Vecna Technologies®	USA	✓	✓	✓	✓	✓	No peer-reviewed studies
Sentri7 by Wolters Kluwer ®	USA	✓	✓	✓	✓	✓	1 peer-reviewed study

* : The availability and specifics of features like alert systems, dynamic reports (automated, interactive dashboard that continuously aggregates and analyzes real-time clinical data such as antibiotic use, resistance patterns, patient outcomes), dose checking, IV-to-PO switch, and infection prevention and control modules can vary based on the version and customization of the software.

+ : See Appendix B for specific DOIs.

IPC: infection prevention and control; IV: intravenous; PO: per oral; EHR: electronic health record.

CDSS integrated within EHR delivers personalized medical recommendations by analyzing patient data and guidelines to suggest optimal treatments, dosages, and durations. These systems incorporate AMS principles, enabling antimicrobial optimization based on clinical data, alerts for revaluation, and suggestions to avoid unnecessary prescriptions.^10,18^ Such systems could incorporate complex recommendations based on ML models. While CDSS offers benefits like workflow integration and customizable features, implementation requires substantial IT infrastructure and maintenance.^19^ Careful workflow evaluation during development should be conducted to maximize end-user uptake.

### Effectiveness of CDSS in healthcare

Despite the promising potential of CDSS to enhance clinical decision-making, a 2010 systematic review found that these systems resulted in less than a 5% improvement in patients receiving targeted care processes.^20^ Although the number of published trials using CDSS interventions has grown over the past decade, a similar analysis in 2020 yielded comparable results.^21^ Among 122 studies (primarily controlled and randomized interventions), CDSS only increased the proportion of patients receiving desired care by 5.8%, with significant variation across the studies.^21^ In the 30 trials reporting clinical endpoints, CDSS resulted in a median 3% increase in patients achieving guideline-based targets. This modest effect raises questions as to the clinical significance of CDSS interventions, given their implementation complexity and cost. A systematic review of 162 randomized controlled trials examined the features of “effective” systems, defined as those improving primary outcomes or at least 50% of secondary outcomes related to care processes or patient health.^22^ More successful systems tended to offer advice to both practitioners and patients, and require justification for overriding recommendations.^22^

## CDSS process outcomes

Baysari et al conducted a meta-analysis of 45 studies evaluating CDSS for AMS, showing increased appropriate antimicrobial use in hospitals (pooled Relative Risk [RR]: 1.49, 95% Confidence Interval [CI]: 1.07–2.08).^23^ However, study quality was suboptimal, and only four types of computerized AMS interventions were included. Analysis of high-quality studies showed inconclusive effects on prescribing (pooled RR: 1.53, 95% CI: .96–2.44), with little evidence of impact on mortality or hospital stay duration. The diverse study designs and outcome measures complicated comparisons between IT interventions. Rawson et al analyzed 58 studies of 38 CDSS for AMS, finding moderate to high-quality evidence that CDSS impact antimicrobial prescribing behaviors.^8^ Outcomes varied from guideline appropriateness to empirical treatment aligned with microbiological sensitivity. The review noted only two randomized controlled trials, one outpatient and one hospital-based.^8^

## CDSS clinical outcomes

Van Dort *et al.* conducted a qualitative synthesis of eight systematic reviews, revealing a trend toward reduced antimicrobial use and improved prescribing appropriateness when using CDSS interventions.^24^ However, significant heterogeneity in interventions and outcome measures prevented meta-analysis. The synthesis reported inconsistent findings regarding the impact of digital interventions on clinical outcomes.^17^ Additional reviews support the potential of CDSS in hospital settings and emphasize the need for higher-quality studies and inclusion of clinical outcomes among patients, including mortality.^25^ A systematic review of 45 studies by Rittman *et al.* found that the efficacy of CDSS and patient outcomes fluctuated based on different settings, underscoring the need for context-specific CDSS that are optimally designed and implemented.^26^ In the context of perioperative prophylaxis, Simpao*et al*. conducted a systematic review of 25 studies and found strong evidence that CDSS could improve antibiotic prescribing practices.^27^ A separate scoping review highlighted the potential of CDSS in pharmacies to decrease antibiotic use in community settings, provided that they are implemented appropriately.^28^

These findings indicate that CDSS show stronger benefits for process outcomes like prescribing appropriateness and reduced antibiotic use, rather than clinical endpoints. The diverse interventions and varying study quality limit comparisons, with modest effect sizes reported.

Schweitzer *et al.* conducted a comprehensive review of studies from 1950 to 2017, evaluating AMS interventions in various settings to identify factors limiting the applicability of research findings to clinical practice.^29^ The review found that most AMS studies were of poor quality, lacking proper control groups and relying heavily on before-after designs (675/825, 82%). While common in quality improvement initiatives, these quasi-experimental studies are prone to bias, leading the Effective Practice and Organisation of Care (EPOC) to discourage their inclusion in systematic reviews in the absence of interrupted time series analyses. In addition, studies investigating AMS intervention in acute care settings report only clinical and microbiological outcomes, frequently omitting key process indicators.

When evaluating AMS interventions, De Kraker et al., highlighted that implementation occurs in complex healthcare environments where biases and temporal effects can obscure causal inferences.^30^ Key challenges include concurrent infection prevention strategies, regression to the mean, and contamination from cluster-level interventions.^30^ These complexities underscore the need for comprehensive measurement of CDSS effectiveness in AMS. A rigorous evaluation framework would enable better understanding of CDSS impact across healthcare settings. Schweitzer et al proposed guidelines to enhance AMS research design, addressing methodology, timing, data gathering, analysis, outcomes, and reporting. The group emphasized aligning study design with goals, such as determining superiority or non-inferiority, and defining effect sizes. Timing should match study objectives, while sequential data collection is crucial for interrupted time series analyses. To minimize bias, suggested approaches include the Desirability of Outcome Ranking (DOOR) and Response Adjusted for Days of Antibiotic Risk (RADAR). Outcomes should cover clinical, microbiological, and care-related areas. They emphasized transparent reporting of effects and suitable controls for practical application.^31^ The prevalence of before-after study designs likely stems from financial constraints and logistical difficulties in conducting controlled trials in healthcare settings. Addressing this requires enhanced support for AMS research and increased methodological capacity to prioritize more rigorous study designs.

Overall, the paucity of high-quality and comparable data remains a major obstacle to assess CDSS effectiveness in AMS, particularly regarding the impact of CDSS on AMR and other microbiological outcomes. Results from quasi-experimental AMS studies should be interpreted cautiously, given the inherent challenges in evaluating CDSS effectiveness in this complex field. These collective findings suggest that while CDSS shows promise in improving antimicrobial prescribing practices, the heterogeneity in study designs, quality, and settings warrants cautious interpretation and highlights the need for further high-quality research to establish their effectiveness. Principal challenges related to assessing effectiveness of CDSS in AMS and are summarized in Table 3.

**Table 3.** Overview of principal challenges to CDSS in AMS

A. Obstacles in assessing the efficacy of CDSS			
Category	Challenge	Impact	Suggested solutions
**Study design** ^8,17,29^	Heterogeneous CDSS types and outcomes	Limits data pooling, weakens conclusions	Define core outcome sets; typologize CDSS
**Methodological quality** ^29,30^	Reliance on before-after studies	High bias, overestimates effect	Use cluster-RCTs or ITS with control; follow EPOC
**Clinical outcome gaps** ^23,25^	Few studies on mortality, AMR, costs	Weak insight on safety and long-term impact	Include clinical, economic, microbiological outcomes
**Validation gaps** ^42,44^	Mostly retrospective, single-site ML-CDSS	Poor generalizability, low trust	Require prospective, multi-center validation
**Evaluation frameworks** ^31,36^	Inconsistent methods/reporting	Poor comparability	Adopt CONSORT-EHEALTH, DOOR, RADAR, TIDieR

B. Challenges in implementing CDSS in clinical practice			
Category	Challenge	Impact	Suggested solutions
**User adoption** ^32,34^	Low uptake (<35%) despite availability	Reduced impact at scale	Engage users; match to clinical needs
**Perceived time burden** ^32^	Belief CDSS slows workflow	Avoidance despite evidence	Time-motion studies, feedback loops
**Workflow integration** ^33,38^	Poor fit in emergencies/clinical flow	Bypassing tools in urgent care	Context-specific adaptation, flexibility
**Alert fatigue** ^39^	Too many/irrelevant alerts	Ignored prompts, loss of trust	Minimizealerts; use relevance tiers
**User engagement and design** ^19,40^	Poor UI, lack of onboarding	Low satisfaction and usage	UX testing, co-design with clinicians
**Trust and interpretability** ^11,41^	Opaque ML logic	Low confidence, accountability issues	Ensure explainable AI, visual rules
**Legal and ethical issues** ^41^	Unclear liability/data risks	Adoption hesitancy by institutions	Clear regulations and legal guidance

ML: machine learning; AI: artificial intelligence.

### Challenges in implementing CDSS for AMS

#### Adoption and uptake

The COMPASS trial^32^ demonstrates challenges in implementing CDSS interventions for AMS. This open-label, cluster randomized superiority trial aimed to reduce antibiotic exposure in hospitalized patients through a computerized AMS intervention. The trial failed to achieve its objective due to low CDSS adoption, being used in only one in four admissions involving antimicrobial prescriptions, with significant implementation delays (average 8.9 d). The non-mandatory prescription re-evaluation meant this step was often ignored, despite high baseline prescribing behaviors.^32^ Similar challenges emerged in the Tanzanian ePOCT+ trial, where Tan et al evaluated a mobile-based CDSS for managing acutely ill children under 15 years in primary care settings. While the intervention decreased antibiotic prescriptions without compromising safety, system adoption remained low, with 25% of patients not managed using the CDSS.^33^

A systematic review and meta-analysis by Kouri *et al.* examined 55 studies (randomized, non-randomized, and quasi-experimental trials that focused on CDSS interventions) and found that only 12.4% reported the adoption or uptake of interventions. When documented, the overall adoption rate was only 34.2%.^34^ This highlights a major barrier to demonstrating the impact of CDSS, as adoption is a critical determinant of real-world effectiveness. According to established implementation science frameworks, uptake by clinicians is essential for translating interventions into meaningful clinical outcomes.^35^ While some studies suggest that partial adoption may still improve prescribing practices, the consistently low adoption rate greatly limits the real-world impact of CDSS interventions.

As many clinical studies of informatics-based interventions omit indicators on adoption or uptake of interventions, the CONSORT-EHEALTH guideline has been developed to enhance the reporting and implementation of these studies.^36^ These findings underscore the complex issues in implementing and assessing CDSS in AMS (Table 3).

#### Workflow integration

While CDSS has the potential to significantly improve antimicrobial prescribing practices, the effectiveness of the interventions depends on factors such as user uptake and engagement. Effectively designing and implementing a digital intervention to ensure the largest uptake by end-users is essential. Well documented problems with usability and widespread dissatisfaction among clinicians using EHR contribute negatively to CDSS uptake and, as a result, limit their effectiveness. Based on the experience of the COMPASS trial, we have shared several considerations on the development, implementation and monitoring of a CDSS for antimicrobial prescriptions from a clinician-researcher point of view.^37^ Key factors include beginning with a well-defined algorithm structure, engaging multidisciplinary teams with clinical and technical experts, and creating user-focused interfaces that clearly demonstrate the logic of guidelines. Finding the right balance between mandatory (“force”) and advisory (“persuasion”) components is also important, as overly strict prompts could lead to resistance, while non-mandatory features could be ignored. Successful implementation was found to rely on usability testing, customized training, and smooth integration with EHR systems. Ultimately, the adoption was affected by how well the CDSS fit into clinical workflows, especially in urgent situations, highlighting that technological capabilities must be paired with careful consideration of the clinical context and the needs of end-users.^37^ Effective systems address both practitioners and patients, requiring justification to override recommendations—characteristics that enhance accountability and adoption.^22^

#### Human factors

Studies using qualitative or mixed approaches have explored ideal characteristics of IT tools supporting antimicrobial prescribing and factors affecting adoption. Key criteria are speed, ease of use, effectiveness and impact.^19,24^ A systematic review by Westerbeek et al of 63 studies identified 327 barriers and 291 facilitators, with factors most related to usefulness of information and system efficiency.^38^

A major implementation challenge is perceived intervention time. In the COMPASS trial, physicians reported that extra time for system use caused dissatisfaction, and informal discussions suggested this perception led prescribers to avoid the system.^32^ However, our timed evaluation using clinical vignettes showed antimicrobial prescription through COMPASS CDSS generally required no additional time compared to standard CPOE.^32^ This demonstrates that perceived extra tasks may limit CDSS adoption even when no actual time burden exists, highlighting the challenge of convincing physicians of such interventions’ value.

A key challenge in implementing CDSS for AMS is alert fatigue, where clinicians become less responsive to excessive alerts, particularly repeated ones.^39^ This is often the main reason for dissatisfaction with current decision support systems. The issue can be addressed through regular evaluation of trigger rules and performance records. Evidence shows that consistent design, appropriate visual presentation of data, controlled terminology, and workflow-integrated advice at the point of decision-making reduce cognitive effort and CDSS mastery time.

Users, accustomed to high-performance technology on their personal devices, expect similar capabilities from any tools intended for professional use. During development, a process known as ‘User Experience (UX)’ should be employed which involves gradual refinement of the tool’s configuration and design to best address user needs and expectations. Although costly, this phase of close collaboration with end-users during development is now considered crucial by developers.^40^

Finally, CDSS are increasingly using artificial intelligence and machine learning approaches. However, there are important ethical and legal considerations that need to be addressed when they are used clinical practice, as well as workflow integration, output transparency challenges, and ensuring adequate training and technical support.^41^

## Conclusions

Effective AMS interventions remain critical to address the complex public health issue of AMR. Although initiatives to improve antimicrobial use have shown potential, achieving lasting changes in prescription behaviours remains difficult. Overcoming entrenched habits and beliefs among prescribers requires a careful analysis of previous interventions with a focus on understanding barriers to success and identifying key drivers of effective change.

Novel strategies, such as CDSS, theoretically offer ways to improve prescribing practices by providing immediate, evidence-based guidance. However, challenges related to integration into clinical workflows, usability, clarity of outputs, data quality, external validation, and interpretability continue to limit their adoption. Moreover, evidence on their real-world effectiveness remains limited, underscoring the need for further high-quality research to establish their impact.Despite the availability of AMS research guidelines for over five years, their limited adoption may reflect several barriers, including insufficient dissemination, competing research priorities, methodological challenges in implementation science, and limited funding dedicated to AMS research.

Future research should prioritize user-centered design, scalable integration into healthcare systems, efforts to facilitate uptake and evaluation frameworks to improve the adoption of effective CDSS in clinical settings.

## Appendix

**Appendix A: URLS for CDSS tools**

**Stand-alone CDSS Tools**

FirstLine Clinical Decision®: www.firstline.org

Antibioclic®: www.antibioclic.com

Sanford Guide AMS®: www.sanfordguide.com

Johns Hopkins ABX Guide®: www.hopkinsguides.com

UpToDate®: www.uptodate.com

ClinicalKey®: www.clinicalkey.com

DynaMed®: www.dynamed.com

**Integrated CDSS Tools**

Nosotech®: Antibiokos: https://nosotech.com/

Epic®: EASM: https://www.epic.com/

Oracle Health®: https://www.oracle.com/health/

TheraDoc®: https://www.theradoc.com/

Vecna QC Pathfinder®: https://vecnahealthcare.com/platform/clinical-surveillance/

Sentri7®: https://www.wolterskluwer.com/en/solutions/sentri7-clinical-surveillance

**Appendix B: DOIs for Scientific Evidence on CDSS Tools**

**Stand -alone tools:**

- FirstLine Clinical Decision®:

10.1016/j.cmi.2025.02.026

10.1371/journal.pone.0252407

10.1017/ice.2022.286

- Antibioclic®:

10.1093/jac/dkaa167

10.2196/60535

10.1016/S1473-3 099(22)00356–5

- Sanford Guide AMS®:

No peer-reviewed studies available.

- Johns Hopkins ABX Guide®:

No peer-reviewed studies available.

- UpToDate®:

10.1002/jhm.944

10.1111/j.1525–1 497.2004.30306.x

10.1007/s11606-007–0206–4

- ClinicalKey®:

10.3163/1536–5 050.101.2.011

- DynaMed®:

10.5195/jmla.2021.1176

10.1055/s-0041–1 742 216

Integrated CDSS tools:

- Nosotech®: Antibiokos:

No peer-reviewed studies available.

- Epic®: EASM:

10.1016/j.ijantimicag.2023.106787

10.1093/ofid/ofx163.580

10.1093/ofid/ofae005

10.1093/ajhp/zxab222

- Oracle Health®:

10.2196/23961

10.1093/jamiaopen/ooab120

10.1017/ice.2022.197

- TheraDoc®:

10.1086/674849

10.1001/jama.294.18.2305

- Vecna QC Pathfinder®:

No peer-reviewed studies available.

- Sentri7®:

10.3390/pharmacy4040032
